# ASM and the UN SDG Publishers Compact: year one

**DOI:** 10.1128/msphere.00116-25

**Published:** 2025-03-17

**Authors:** Lorraine F. Clark, Amanda Donaldson, Michael E. Lerman

**Affiliations:** 1 American Society for Microbiology 11003, Washington, DC, USA

**Keywords:** sustainability, sustainable development goals, SDGs, publishing

## Abstract

The United Nations (UN) Sustainable Development Goals (SDG) represent an important set of global priorities, addressing the most urgent environmental, economic, and social challenges facing humankind. The American Society for Microbiology (ASM) signed the UN SDG Publishers Compact in March 2024, to lend its voice to this vital initiative, as indeed not only do the SDGs align with ASM’s mission but microbes themselves can play significant roles in, for example, sustainable agriculture, clean energy, and human health. In its first year as a signatory, ASM has pursued, published, and highlighted sustainability-focused work in its journals, has raised awareness through internal efforts and joint efforts with other scientific organizations, and has prioritized the SDGs in its conferences and other programs. We commemorate our achievements in this first year and look forward to future initiatives, innovations, and collaborations toward a sustainable future.

## EDITORIAL

How can a publisher help save the world? How can a professional society focused on microbiology make an impact on grand challenges like poverty, inequality, economic crises, and environmental disasters? The questions may be complicated—they certainly are daunting—but we are committed to answering them. A sustainable future relies on our collective capacity and will to achieve it.

In March 2024, the American Society for Microbiology (ASM) signed the United Nations Sustainable Development Goals (UN SDG) Publishers Compact (https://asm.org/Press-Releases/2024/March/ASM-Joins-UN-Sustainable-Development-Goals-Publish). This was both a recommitment to advancing the microbial sciences toward global sustainability and a promise to reflect, focus, and consider new opportunities.

ASM was established in 1899 and is one of the largest professional life science organizations worldwide. Its journals have been in the vanguard of scientific publishing for over a century, and other departments within the organization, such as Public Policy and Advocacy, the Global Public Health Program, Education, Meetings, and the American Academy of Microbiology, have worked tirelessly with members and stakeholders worldwide to tackle some of humanity’s greatest challenges.

The UN SDGs were initially developed in 2015 as part of the 2030 Agenda for Sustainable Development, as a road map for global peace and prosperity, addressing elements like quality education, gender equality, affordable and clean energy, and responsible consumption and production. These topics are diverse, requiring efforts across disciplines and industries, but they all relate to a secure future for our world. What is more, they also relate to the microbial sciences, either in the myriad ways that microbes impact humankind or in the ways we teach, learn, study, and conduct research into microbiology.

Signing the Compact was an easy decision, in line with ASM’s vision to “proactively harness the power of microbes to solve humanity’s most pressing challenges” (https://asm.org/About-ASM/Charting-ASM-s-New-Strategic-Course). Equally important are ASM’s mission and values (https://asm.org/about-asm), which align with SDGs such as “Gender Equality” and “Reducing Inequalities.” To that end, fulfilling the Compact was a work already in progress but one to which we dedicated renewed efforts and new strategies.

ASM journals have individually sought and promoted research reflecting the SDGs. For instance, the *Journal of Microbiology & Biology Education* (JMBE) has had two special series recently, 2023–2024’s “Teaching Climate Change” (centering on both “Climate Action” and “Quality Education”) and 2024–2025’s “Teaching Assistant Professional Development in STEM” (focused on the teacher training aspects of “Quality Education”). Another example is *Microbiology Spectrum*’s Diagnostic Testing and Laboratory Equity in Clinical Microbiology series, to support global initiatives for equitable access to clinical diagnostics (“Good Health and Well-Being,” “Reducing Inequalities”). Similarly, *Infection and Immunity* (IAI) is conducting a call for papers on Women’s Health and the Development of Microbiota in Childhood, to address important SDG targets related to the reduction of both maternal mortality and neonatal mortality. We have seen encouraging readership of the articles published in these collections so far and will continue to support research and perspectives on such important topics. ASM is not taking these steps alone. *mSphere* partnered with the AGU journal *GeoHealth* for a joint call for papers. This special series, One Health, Microbes, and Climate Change, covers environmental and socially relevant SDGs, underscoring their interconnectivity in both the problems humanity faces and their potential solutions. As well, in November 2024, *mSystems* co-published a widely publicized editorial on the “climate catastrophe” with several other publishers (including other SDG Publishers Compact signatories). These initiatives reflect ASM’s commitment to collaboration and using our platform to advance sustainability-focused efforts.

In September 2024, to coincide with the UN General Assembly (and Sustainability Summit), we posted the inaugural ASM Journals SDG Spotlight Collection, which featured notable articles aligned with the SDGs and saw enthusiastic engagement on social media platforms. In the same month, multiple departments collaborated for a revision of ASM’s The Role of Microbiology in Sustainable Development article, including recent developments across the field and the organization, such as advocacy for antimicrobial stewardship and efforts to increase laboratory capacity in low- and middle-income countries. Interdepartmental collaboration within ASM is a vital part of the approach to the Compact, to both increase our collective awareness about sustainable strategies and innovations (e.g., sharing articles, explainers, and online resources) and identify further areas for cooperation and innovation.

The Compact goes beyond just the printed (or digital) page, and ASM has pursued sustainable solutions and partnerships in other arenas. For example, many of ASM’s partners and vendors have comprehensive climate/environmental impact reports or corporate responsibility statements, and most of ASM’s upcoming meetings will be held at LEED Gold Certified venues. Moreover, at ASM’s Microbe conference in June 2025, numerous sessions based on both sustainability in microbiology and the SDGs themselves have been planned, as well as several SDG poster prizes, and we look forward to showcasing these efforts at the event. Altogether, we have unique opportunities to support the SDGs both in word and in deed, and we commit to continue pursuing them.

We are proud of our accomplishments in this first year since signing the Compact, but there is challenging work yet to do in the months and years ahead, catalyzing our actions in the publication field into meaningful change in the world. We look forward to celebrating the progress being achieved and promoting research into fields where progress is still needed, through our journals, through our meetings, indeed, through our organization at large. We will keep pursuing work that can accelerate essential innovations and inform life-improving public policies. We stand alongside our fellow publishers, researchers, educators, and all in the microbiology community ready to continue the journey toward the ultimate goal of a sustainable future.

